# Supplementary material for: Sigma-1 Receptor (S1R) Interaction with Cholesterol: Mechanisms of S1R Activation and Its Role in Neurodegenerative Diseases
Source: Int J Mol Sci. 2021 Apr 15;22(8):4082. doi: 10.3390/ijms22084082 (PMC8071319; doi:10.3390/ijms22084082)
Supplement: Supplementary file 1 [file ijms-22-04082-s001.zip › ijms-1178267-SI NEW/Supplementary Tables/Supplementary table S3.pdf]

Supplementary table 3. Cell surface proteins and receptors

Gene	Name	Fold enrichment	P-value
CD109	CD109 molecule	11.0	0.001
ITGA1	Integrin subunit alpha 1	3.1	0.001
ITGAV	Integrin subunit alpha V	20.5	0.001
NRP1	Neuropilin 1	4.2	0.001
PCSK9	Proprotein convertase subtilisin/kexin type 9	10.9	0.001
TRABD2A	Metalloprotease TIKI1	16.5	0.001
SEMA3B	Semaphorin-3B	4.8	0.001
OSMR	Oncostatin-M-specific receptor subunit beta	3.4	0.001
CNTNAP1	Contactin-associated protein 1	35.0	0.003
ADAM10	ADAM metallopeptidase domain 10	9.7	0.004
APLP2	Amyloid-like protein 2	5.7	0.004
NOTCH2	Notch 2	2.6	0.005
ADAM9	ADAM metallopeptidase domain 9	12.2	0.008
CACNA2D1	Voltage-dependent calcium channel subunit alpha-2/delta-1	19.6	0.008
EGFR	Epidermal growth factor receptor	4.8	0.009
ITGB1	Integrin subunit beta 1	5.2	0.01
ITGB5	Integrin subunit beta 5	4.2	0.01
LRRC8A	Leucine rich repeat containing 8 family member A	3.2	0.01
LRRC8A	Volume-regulated anion channel subunit LRRC8A	3.2	0.01
IGF2R	Insulin like growth factor 2 receptor	2.6	0.02
PLXNB2	Plexin B2	11.0	0.02
PVR	Poliovirus receptor	5.0	0.02
SCARB1	Scavenger receptor class B member 1	9.4	0.02
LBR	Lamin-B receptor	4.6	0.02
ADAM15	ADAM metallopeptidase domain 15	16.2	0.03
APP	Amyloid beta precursor protein	6.7	0.03
SLC39A6	Solute carrier family 39 member 6	9.6	0.03

SLC39A6	Zinc transporter ZIP6	9.6	0.03
CTNND1	Catenin delta-1	3.0	0.03
L1CAM	L1 cell adhesion molecule	10.6	0.04
ITGA3	Integrin subunit alpha 3	7.3	0.04
ITGA5	Integrin subunit alpha 5	9.0	0.04
AXL	AXL receptor tyrosine kinase	2.7	0.05
LRPAP1	LDL receptor related protein associated protein 1	17.3	0.05
HM13	Histocompatibility minor 13	5.2	0.05
NEGR1	Neuronal growth regulator 1	4.2	0.05
