# Supplementary material for: Sigma-1 Receptor (S1R) Interaction with Cholesterol: Mechanisms of S1R Activation and Its Role in Neurodegenerative Diseases
Source: Int J Mol Sci. 2021 Apr 15;22(8):4082. doi: 10.3390/ijms22084082 (PMC8071319; doi:10.3390/ijms22084082)
Supplement: Supplementary file 1 [file ijms-22-04082-s001.zip › ijms-1178267-SI NEW/Supplementary Tables/Supplementary table S2.pdf]

Supplementary table 2. Extracellular matrix component

Gene	Name	Fold enrichment	P-value
NENF	Neudesin	10.6	0.0003
LAMB2	Laminin subunit beta 2	13.5	0.001
MANF	Mesencephalic astrocyte-derived neurotrophic factor	80.6	0.001
GPC1	Glypican-1	7.8	0.001
LAMB1	laminin subunit beta 1	17.5	0.002
RXDN	Peroxidasin	6.5	0.002
SULF2	Extracellular sulfatase Sulf-2	12.8	0.002
GALS3BP	Galectin 3 binding protein	2.7	0.003
TGFBI	Transforming growth factor beta induced	16.0	0.003
LGALS3BP	Galectin-3-binding protein	2.7	0.003
LAMC1	Laminin subunit gamma 1	7.4	0.008
COL6A1	Collagen type VI alpha 1 chain	7.6	0.01
BCHE	Cholinesterase	3.1	0.01
COL12A1	collagen type XII alpha 1 chain	51.0	0.016
AGRN	Agrin	5.6	0.02
MYDGF	Myeloid-derived growth factor	109.9	0.02
CFH	Complement factor H	13.4	0.02
FBLN1	Fibulin 1	3.6	0.027
GPC5	Glypican-5	10.3	0.03
BMP1	Bone morphogenetic protein 1	5.8	0.03
FBLN1	Fibulin-1	3.6	0.03

COL7A1	Collagen type VII alpha 1 chain	16.2	0.04
TGFB2	Transforming growth factor beta 2	3.4	0.04
SAP	Prosaposin	36.0	0.04
FUCA2	Plasma alpha-L-fucosidase	29.4	0.04
COL5A1	Collagen type V alpha 1 chain	14.6	0.05
